# Strength Training and Testosterone Treatment Have Opposing Effects on Migration Inhibitor Factor Levels in Ageing Men

**DOI:** 10.1155/2013/539156

**Published:** 2013-09-08

**Authors:** D. Glintborg, L. L. Christensen, T. Kvorning, R. Larsen, K. Brixen, D. M. Hougaard, B. Richelsen, J. M. Bruun, M. Andersen

**Affiliations:** ^1^Department of Endocrinology, Odense University Hospital, 5000 Odense C, Denmark; ^2^Institute of Sport Science and Clinical Biomechanics, University of Southern Denmark, 5000 Odense, Denmark; ^3^Department of Informatics and Mathematical Modelling, Technical University of Denmark, 2800 kongens Lyngby, Denmark; ^4^Department of Clinical Biochemistry and Immunology, Statens Serum Institut, 2300 Copenhagen S, Denmark; ^5^Department of Internal Medicine and Endocrinology (MEA), Aarhus University Hospital, 8000 Aarhus, Denmark; ^6^Medical Department, Regional Hospital Randers, 8900 Randers, Denmark

## Abstract

*Background.* The beneficial effects of testosterone treatment (TT) are debated. *Methods.* Double-blinded, placebo-controlled study of six months TT (gel) in 54 men aged 60–78 with bioavailable testosterone (BioT) <7.3 nmol/L and waist >94 cm randomized to TT (50–100 mg/day, *n* = 20), placebo (*n* = 18), or strength training (ST) (*n* = 16) for 24 weeks. Moreover, the ST group was randomized to TT (*n* = 7) or placebo (*n* = 9) after 12 weeks. *Outcomes*. Chemokines (MIF, MCP-1, and MIP-1**α**) and lean body mass (LBM), total, central, extremity, visceral, and subcutaneous (SAT) fat mass established by DXA and MRI. *Results*. From 0 to 24 weeks, MIF and SAT decreased during ST + placebo versus placebo, whereas BioT and LBM were unchanged. TT decreased fat mass (total, central, extremity, and SAT) and increased BioT and LBM versus placebo. MIF levels increased during TT versus ST + placebo. ST + TT decreased fat mass (total, central, and extremity) and increased BioT and LBM versus placebo. From 12 to 24 weeks, MCP-1 levels increased during TT versus placebo and MCP-1 levels decreased during ST + placebo versus placebo. *Conclusion*. ST + placebo was associated with decreased MIF levels suggesting decreased inflammatory activity. TT may be associated with increased inflammatory activity. This trial is registered with ClinicalTrials.gov NCT00700024.

## 1. Introduction

Testosterone replacement therapy is indicated in severe hypogonadism, whereas the indication for testosterone therapy (TT) in aging hypogonadal men without hypothalamic, pituitary, or testicular disease is debated [1, 2]. In particular, the effects of TT on metabolic and cardiovascular outcomes are undetermined [3]. Recently, we reported that TT increased lean body mass (LBM) and lipid oxidation [1], whereas lower extremity fat mass (LEFM) and abdominal subcutaneous adipose tissue (SAT) decreased, but visceral adipose tissue (VAT) was unchanged [4]. In addition to these testosterone-induced changes in body composition and lipid metabolism, we observed decreased levels of high density lipoprotein, adiponectin, and osteoprotegerin [5], whereas the cardiovascular risk markers, LDL, c-reactive protein, and insulin sensitivity measured by clamp were unchanged [1, 4]. Recent meta-analyses found no effect of  TT on cardiovascular outcomes, but the duration of the included studies was limited and cardiovascular outcomes were not reported in all trials [6]. The overall interpretation of the combined effects of TT on metabolic and cardiovascular risk factors remains undetermined, and additional studies are needed.

Adiposity is a major component of the metabolic syndrome and an independent risk factor for the development of type 2 diabetes and cardiovascular disease [7]. Human adipose tissue produces and releases a number of bioactive proteins, collectively referred to as adipokines [8]. Adipokines are primarily secreted by adipose tissue-resident macrophages [9]. In obesity, the number of adipose tissue-resident macrophages is increased in both SAT and VAT [10, 11], and circulating mononuclear cells are in a more inflammatory state [12]. Chemokines are an important subgroup of adipokines, which activate (chemoattract) mononuclear cells during the process of inflammation. Increasing adiposity is associated with influx of monocytes to the AT with concomitant differentiation of AT-resident macrophages from a primarily anti-inflammatory to a proinflammatory state [10]. Interestingly, the influx of monocytes may be orchestrated by several chemokines. Monocyte chemoattractant protein-1 (MCP-1) and migration inhibitor factor (MIF) are positively associated with obesity [13–15]; these chemokines have the ability to induce insulin resistance [16] and are predictors for type 2 diabetes [17]. Obesity is inversely associated with testosterone levels [18], but the effect of TT on chemokine levels remains to be established. 

Aging men with low normal testosterone levels are often characterized by decreased LBM, decreased muscle strength, and relative inactivity [19]. Physical exercise is associated with decreased chemokine levels [13, 31]. We are not aware of previous studies evaluating the effect of combined testosterone and strength exercise on chemokine levels in ageing men.

In the present study, we investigated the effects of TT on chemokine levels in aging men with low normal testosterone levels [18] and increased waist circumference. We hypothesized that decreased SAT during TT could be associated with decreased chemokine levels. We furthermore examined if strength exercise with and without TT was associated with decreased chemokine levels.

## 2. Methods

The study was a single center, randomized, placebo-controlled, six-month study to assess the effect of testosterone gel and strength training (ST) on body composition, components of the metabolic syndrome, and quality of life in men with low normal bioavailable testosterone (BioT) levels and increased body fat. The inclusion criteria for participation in the study were age 60–78 years, BioT <7.3 nmol/L, and waist circumference >94 cm. The exclusion criteria were hematocrit >50%, prostate cancer or a prostate specific antigen >3 ng/dL, previous or ongoing malignant disease, severe ischemic heart or respiratory disease, disability, diabetes mellitus, alcohol or drug abuse, abnormal routine blood samples (TSH, ionized calcium, hemoglobin, and liver and kidney functions), and treatment with 5*α* reductase inhibitors, morphine, or oral glucocorticoid steroids. 

The sample size of the study was determined by the effect of TT on LBM and has been described previously [1]. LBM was chosen as the primary study outcome based on a meta-analysis on testosterone therapy in ageing men by Isidori et al. [20]. Chemokine levels were secondary study outcomes. 

**Figure 1:**
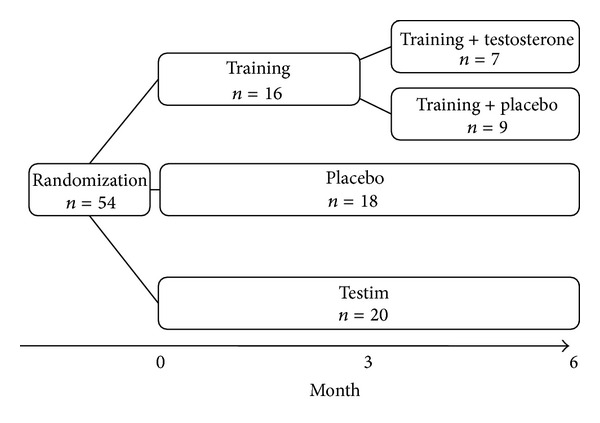
Study design.

Subjects were randomly assigned to receive testosterone (TT, *n* = 20), placebo (*n* = 18), or engage in strength training (*n* = 16) as previously described [1]. After 12 weeks, the ST group was randomized into two groups receiving testosterone or placebo (ST + TT, *n* = 7, and ST + placebo, *n* = 9) (Figure 1). Treatment with testosterone and placebo was double blinded. Randomization numbers were assigned to the participants in order of enrollment into the study. The study was approved by the local Ethics Committee and declared in http://www.clinicaltrials.gov/ (NCT00700024). All participants gave written informed consent. Exclusion criteria were hematocrit >50%, prostate cancer or a prostate specific antigen (PSA) >3 ng/dL, previous or ongoing malignant disease, severe ischemic heart or respiratory disease, disability, diabetes mellitus, alcohol or drug abuse, abnormal routine blood samples (TSH, ionized calcium, hemoglobin, and liver and kidney functions), and treatment with 5*α* reductase inhibitors, morphine, or oral glucocorticoid steroids. Four patients had previous apoplexia cerebri, and three patients had ischemic heart disease. None of the study participants were restricted during daily living activities. A total of 42% of participants were on antihypertensive drugs, 29% were on cholesterol-lowering drugs, 10% were on inhalation steroids, 6% were on antidepressants, and 4% were treated for enlarged prostate. The distribution of concomitant medication in the treatment arms was equal. No cholesterol-lowering drugs or other medications were introduced during the study. Participants received 5–10 g gel/50–100 mg testosterone (Testim, Ipsen, France) or 5–10 g gel/placebo. The randomization list, medicine labeling, and randomization and code break envelopes were generated by Ipsen Scandinavia (Kista, Sweden) to ensure double blinding. Compliance was monitored by participant self-reporting at each visit. The study outcomes were evaluated at baseline and after three and six months' intervention.

### 2.1. Training Protocol

The training protocol has been described recently [21]. In brief, participants performed 5 min bicycling for warm up (approximately 100 W) followed by a progressive heavy strength training program including exercises for the entire body and gradually increased training loads [21]. All training sessions were supervised, and patients and supervisors were blinded for placebo/testosterone treatment. All subjects participated in minimum 2 out of 3 weekly training sessions (mean training adherence 75 ± 8%). Subjects were advised to refrain from self-initiated resistance exercise training and intense endurance training but were allowed to continue on other habitual activities throughout the study. All subjects received 0.2 liter of skimmed chocolate milk (containing 7 g protein, 20 g carbohydrate, and 1 g fat) after each strength training session, but in addition, subjects were informed not to change their diet.

### 2.2. Biochemical Analyses

#### 2.2.1. Chemokines

Plasma levels of the investigated chemokines were assessed using a specific human enzyme-linked immunosorbent assay (ELISA) method (DuoSet, R&D Systems Europe Ltd., UK). MCP-1, macrophage inflammatory protein- (MIP-) 1*α*, and MIF assays had intra-assay coefficient of variation (CV) of 8.1% (*n* = 12), 7.1% (*n* = 12), and 4.6% (*n* = 12), respectively. The samples used for the chemokine analyses had not previously been thawed.

#### 2.2.2. Testosterone and SHBG

Testosterone was measured after an overnight fast between 8 and 9 AM. Serum total testosterone was measured by liquid chromatography tandem mass spectrometry after ether extraction. For testosterone measurements, the intra-assay coefficient of variation was 10% for total testosterone >0.2 nmol/L and 30% in the range between 0.1 and 0.2 nmol/L. SHBG was measured by autoDELFIA assay, and BioT was calculated according to the formulas of Vermeulen et al. [22], http://www.issam.ch/freetesto.htm. During calculations, we used the assumption that albumin concentration in participants was 4.3 g/L. A single measurement of testosterone was performed to determine eligibility. BioT levels were all below 7.3 nmol/L at reevaluation after three weeks on placebo treatment (*n* = 18). The normal range and 95% confidence interval for BioT were 7.3 nmol/L (7.0–7.5 nmol/L) [18].

### 2.3. Body Composition Measures

#### 2.3.1. Dual X-Ray Absorptiometry (DXA)

TFM, central fat mass (CFM), lower extremity fat mass (LEFM), and LBM were measured by DXA using a Hologic Discovery device (Waltham, MA, USA). The CV was 0.8% for TFM and 0.6% for LBM, respectively.

#### 2.3.2. Magnetic Resonance Imaging

Magnetic resonance imaging was performed as previously reported [4]. In brief, a 3.0 Tesla High field MR Unit was used (Philips Achieva, Phillips Healthcare, Best, The Netherlands). One abdominal slice (10 mm thick, intervertebral space of  L4/L5, perpendicular to subcutaneous fat) was recorded. Computer software was used to trace the different compartments of fat on the abdomen and for assessment of the areas of SAT and VAT. The thigh fat area was determined on one femoral slice (15 cm from the major trochanter and perpendicular to subcutaneous fat) using a T1-weighted gradient-echo sequence (repetition time 400 ms, echo time 18 ms, acquisition matrix 376 × 335, field of view 230 × 230 mm). Computer software was used to trace fat and muscle compartments on the thigh to assess subcutaneous and intramuscular fat and thigh muscle area [23]. Due to various reasons (claustrophobia, did not attend, and did not want examination), complete MRI data were only available for 54 patients. The number of patients not attending first MRI was placebo: 2, TT: 1, ST + placebo: 1, and ST + TT: 2. The number of patients not attending the MRI at study termination was placebo: 2, TT: 1, ST + placebo: 2, and ST + TT: 1. Patients without MRI were included in the data analyses that did not include MRI data.

### 2.4. Statistical Analysis

Pretreatment differences between patients in the testosterone and placebo group were tested using the Mann-Whitney *U* tests. The effect of placebo, TT, ST + placebo, and ST + TT was analyzed by comparing delta (Δ) values of hormonal and metabolic variables using the Mann-Whitney *U* tests. Δ-values were calculated as post treatment level minus pretreatment level of each analyzed variable. In this way, Δ-values were positive if the measured variable increased during intervention. Δchemokine levels were correlated with Δ-values of hormonal and metabolic variables using the Spearman nonparametric correlation tests. 

All statistics were performed using SPSS 17.0 (SPSS Inc., Chicago, USA) for calculations and *P* values < 0.05. were considered significant. Data are given as median and interquartile range.

## 3. Results

### 3.1. Study Population (*n* = 54)

Baseline data are shown in Table 1. There were no significant differences at baseline regarding age, chemokine levels, and body composition. Baseline data on testosterone and body composition in the testosterone and placebo groups have been presented previously [1, 4]. 

### 3.2. Intervention

Changes in chemokines, BioT, and body composition during 3 and 6 months' intervention are shown in Table 2 (effects of TT and ST on BioT and body composition have recently been discussed) [4, 21]. MIP-1 *α* levels and VAT were unchanged during all interventions at 3 and 6 months, VAT data not shown.

TT alone had no significant effects on muscle strength, whereas ST significantly increased muscle strength independent of the addition of testosterone (data not shown, discussed in [21]). 

### 3.3. Six-Month Effects (6–0)

Circulating MIF levels decreased during ST + placebo and were significantly different from changes in MIF during placebo and changes during TT, but not from changes in MIF during ST + TT (Table 2). 

TT increased BioT and LBM versus placebo, and fat mass (TFM, CFM, LEFM, and SAT) decreased. 

ST + placebo decreased fat mass (TFM, LEFM, and SAT) versus placebo without significantly affecting BioT and LBM.

ST + TT was followed by similar BioT and LBM compared to TT alone. The beneficial effects of ST + TT on fat mass measures were comparable to ST + placebo.

### 3.4. Three-Month Effects (3–0)

Chemokine levels were unchanged during the first three months of intervention (Table 2). 

### 3.5. Three-Month Effects (6–3)

ST + placebo versus placebo and versus ST + TT was followed by decreased MCP-1 levels (Table 2).

### 3.6. Bivariate Associations between ΔChemokines and ΔBody Composition Measures ST + TT (6–0)

ΔMIF was significantly associated with Δfat (*r* = −0.83, *P* < 0.05) and ΔLEfat (*r* = −0.86, *P* < 0.05). ΔMIP was significantly associated with Δfat (*r* = −0.93, *P* < 0.001), ΔCFM (*r* = −0.75, *P* < 0.05), and ΔLEfat (*r* = −0.96, *P* < 0.001).

### 3.7. TT and ST + Placebo (6–0)

No significant associations were found between Δchemokine levels and Δfat mass measures during TT or during ST + placebo.

## 4. Discussion

In the present study, MIF levels increased during TT, whereas ST + placebo was associated with decreased MIF and MCP-1 levels in ageing men with low normal BioT and increased waist circumference. Our findings suggest opposing effects of TT and ST on inflammatory status. 

Given the on-going debate on testosterone therapy in aging hypogonadal men without pituitary or testicular disease, it is important to clarify possible favorable or unfavorable effects of testosterone therapy [24]. Our findings of positive associations between testosterone and chemokine levels support previous observations of higher circulating MIF in males compared to females [17, 25] and findings from cross-sectional studies [25]. Patients with polycystic ovary syndrome and hyperandrogenemia showed a BMI and SHBG independent correlation between chemokine levels and testosterone, further supporting that high testosterone levels may increase inflammation markers [26]. In the present study, ΔBioT and Δchemokine levels were not associated, and these data do not support a direct effect of testosterone on inflammatory status. Instead, the effects of TT on chemokine secretion could be indirectly affected by other (inflammatory) pathways or by factors such as fat mass or LBM as discussed below. 

We are not aware of previous studies that evaluated the long-term effect of randomized TT on chemokine levels in ageing male study populations. Six-month DHEAS treatment in women with adrenal insufficiency did not affect MCP-1 levels despite increased LBM [27], but fat distribution was unchanged and MIF data were not available in the study. Interestingly, animal studies showed that rats express MIF in Leydig cells, but whether MIF affects human reproduction is undetermined [25, 28]. Exogenous testosterone could increase MIF levels through a decreased testicular LH stimulation, but future studies are needed to test this hypothesis. The present data along with previous studies therefore support that increased testosterone levels are associated with increased chemokine levels. Increased circulating levels of MCP-1 and MIF are positively associated with the development of various metabolic diseases such as type 2 diabetes, atherosclerosis, and cardiovascular disease [29, 30]. The impact of increased chemokine levels during testosterone treatment on long-term cardiovascular risk awaits long-term studies.

In the present study, ST was associated with reduced MIF and MCP-1 levels suggesting decreased inflammatory activity. These findings support previous reports in which lifestyle intervention with diet and/or increased physical activity decreased circulating chemokine levels, reduced infiltration of macrophages into the AT, and improved whole-body inflammation [31]. We found that MCP-1 levels decreased during the last three months of the intervention period. This could suggest that longer time of exercise is needed to improve inflammatory status perhaps mediated by improved body composition during physical exercise. Chemokine secretion is higher in VAT than in SAT [11, 32] and inflammation is unchanged when the SAT depot is selectively reduced by abdominal liposuction [33]. Our study supports these findings as SAT decreased along with adiponectin during TT [4]. In the present study, however, VAT was unchanged during TT and ST, and the highest decrease in SAT was observed during TT. We found that ΔMIF and ΔMIP were strongly inversely associated with ΔLEFM supporting that a “pear-shape” is associated with decreased inflammatory activity [34, 35] as well as higher adiponectin levels [35]. The strong inverse association between ΔMIF and ΔMIP and central and total fat mass during ST + TT treatment was an unexpected finding in the present study. ΔVAT and ΔSAT were unassociated with Δchemokine levels suggesting that changed fat mass was not the only predictor of chemokine levels. 

In the present study, resistance training and not aerobic training was included in the intervention. A recent study in patients with type 2 diabetes found that aerobic training was associated with similar weight loss but had more beneficial effects on inflammatory markers than ST [36]. Future studies are needed to evaluate possible effects of various exercise interventions on inflammatory status in men during TT.

Change in LBM was our main study outcome [20]. We found that LBM was unaffected by ST alone, whereas TT alone or in combination with ST significantly increased LBM with no additional effect of the addition of testosterone. Our findings of increased LBM independent of muscle strength are in agreement with previous studies and have recently been discussed [21]. We found no significant association between changes in chemokine levels and changes in LBM, suggesting that muscle tissue is not the main regulator of chemokine secretion. Indeed, muscle tissue expresses and releases very low levels of chemokines even in relation to exercise [31].

In conclusion, the findings of the present study support that TT and ST have similar and positive effects on fat mass; however, ST is superior to TT regarding chemokine levels, inflammatory status, and body strength. Further studies on cardiovascular end points are needed to determine the long-term effects of TT alone and in combination with lifestyle intervention in ageing men with low normal BioT.

## Figures and Tables

**Table 1:** Baseline data.

	Placebo (*n* = 18)	TT (*n* = 20)	ST (*n* = 16)
Age (years)	67 (65–70)	68 (62–72)	68 (62–73)
MIF (pg/mL)	203 (128–378)	182 (117–453)	259 (199–498)
MCP-1 (pg/mL)	71 (59–86)	72 (63–105)	87 (64–101)
MIP-1*α* (pg/mL)	27 (13–1981)	17 (9–1257)	29 (11–1764)
BioT (nmol/L)	4.4 (3.3–6.0)	5.1 (4.3–6.1)	5.3 (2.2–6.2)

LBM (kg)	64.7 (58.5–73.3)	64.6 (57.2–71.3)	65.4 (60.2–72.0)
Waist (cm)	105 (98–118)	107 (103–115)	107 (104–115)
Fat mass (kg)	23.9 (18.2–33.1)	24.3 (21.4–31.8)	25.2 (22.5–28.7)
Central fat mass (kg)	13.1 (9.0–18.4)	14.0 (12.5–18.2)	14.7 (12.3–17.4)
SAT	24.2 (21.5–34.9)	29.3 (24.5–40.7)	27.9 (24.6–33.1)
VAT	15.6 (11.5–20.0)	15.1 (12.4–19.3)	16.5 (12.3–19.3)

Data presented as median (interquartile range).

Baseline data from the subgroups ST + placebo and ST + TT can be seen in Table 2.

No significant differences, Mann-Whitney test between groups.

BioT: bioavailable testosterone.

LBM: lean body mass.

SAT: subcutaneous adipose tissue.

VAT: visceral adipose tissue.

**Table 2:** Clinical and biochemical characteristics in patients during testosterone, placebo, and strength training.

			Placebo *n* = 18	TT *n* = 20	ST ST + placebo *n* = 9	ST ST + TT *n* = 7
MIF (pg/mL)	*t* = 0	(3–0)	203 (128–378)	182 (117–453)	266 (118–660)	253 (228–394)
	*t* = 3	(6–3)	205 (136–411)	282 (183–582)	207 (119–704)	277 (239–376)
	*t* = 6	(6–0)*	227 (141–649)	412 (170–777)	129 (101–471)^¤#^	235 (169–301)

MCP-1 (pg/mL)	*t* = 0	(3–0)	71 (59–85)	72 (63–105)	98 (62–109)	85 (59–87)
	*t* = 3	(6–3)*	74 (56–101)	84 (67–107)^#^	93 (70–117)^#^	76 (58–93)^¤§^
	*t* = 6	(6–0)	82 (62–101)	80 (64–100)	72 (58–93)	79 (59–105)

MIP-1*α* (pg/mL)	*t* = 0	(3–0)	27 (13–1981)	17 (8–1257)	13 (8–827)	200 (18–2884)
	*t* = 3	(6–3)	26 (13–1855)	17 (11–1318)	14 (9–798)	263 (18–2390)
	*t* = 6	(6–0)	31 (13–1800)	16 (10–1059)	13 (8–856)	347 (18–2152)

BioT (nmol/L)	*t* = 0	(3–0)*	4.4 (3.3–6.0)	5.1 (4.3–6.1)^##^	5.1 (5.0–6.2)^¤^	5.7 (4.8–6.2)^##§§^
	*t* = 3	(6–3)	4.3 (3.3–4.8)	8.1 (5.6–12.3)	4.5 (4.2–5.0)	4.5 (4.1–5.1)
	*t* = 6	(6–0)**	3.9 (3.4–4.8)	9.7 (6.5–13.8)^#^	4.3 (3.7–4.4)^¤^	12.8 (7.3–16.3)^##§^

LBM (kg)	*t* = 0	(3–0)**	64.7 (58.5–73.2)	64.7 (57.2–71.2)^#^	63.2 (59.8–68.7)	70.9 (65.1–78.2)^##§^
	*t* = 3	(6–3)	65.2 (58.9–73.3)	66.1 (59.9–72.2)	63.2 (57.5–68.9)	73.1 (63.3–78.2)
	*t* = 6	(6–0)*	64.3 (58.8–74.7)	65.8 (59.3–72.6)^#^	63.8 (5.9–6.9)	75.4 (66.0–78.7)^#^

Fat mass (kg)	*t* = 0	(3–0)	23.9 (18.2–33.1)	24.4 (21.4–31.8)	25.0 (20.9–28.9)	25.4 (24.0–27.1)
	*t* = 3	(6–3)	24.3 (18.0–32.1)	23.8 (21.4–31.3)	25.9 (21.5–28.0)	25.9 (22.9–26.3)
	*t* = 6	(6–0)*	25.1 (19.2–31.3)	23.0 (20.3–30.2)^#^	24.2 (21.5–27.8)^#^	24.7 (21.5–25.9)^#^

CFM (kg)	*t* = 0	(3–0)	13.1 (9.0–18.4)	14.0 (12.5–18.2)	13.9 (11.8–17.6)	15.0 (13.7–16.9)
	*t* = 3	(6–3)	13.1 (8.5–18.4)	13.6 (12.2–18.2)	14.5 (12.2–16.5)	14.6 (13.6–15.7)
	*t* = 6	(6–0)*	13.5 (9.2–17.6)	13.9 (11.2–17.4)^#^	12.4 (12.1–15.6)	13.5 (13.1–15.9)^#^

LEfat (kg)	*t* = 0	(3–0)	6.9 (5.6–9.5)	6.5 (5.6–7.9)	7.2 (5.7–8.6)	7.4 (6.4–8.1)
	*t* = 3	(6–3)	7.2 (5.3–9.8)	6.4 (5.5–8.2)	7.2 (5.7–8.4)	6.8 (6.6–7.5)
	*t* = 6	(6–0)*	7.2 (5.8–9.7)	6.1 (5.2–8.0)^#^	6.8 (5.3–8.2)^#^	6.6 (6.5–7.2)^#^

SAT	*t* = 0	(3–0)	24.2 (21.5–34.9)	29.2 (24.5–40.7)	29.6 (24.9–39.7)	27.9 (23.2–28.3)
	*t* = 3	(6–3)*	23.7 (20.8–35.3)	29.7 (24.2–40.1)^##^	30.3 (25.2–39.9)^#^	26.7 (23.4–28.7)^¤§^
	*t* = 6	(6–0)**	25.7 (20.2–36.5)	25.6 (21.7–37.2)^#^	29.7 (24.0–37.4)^#^	26.6 (23.5–31.1)^¤§^

Data presented as median (interquartile range).

Kruskall-Wallis tests followed by Mann-Whitney test performed on delta values.

*P < 0.05 between groups, **P < 0.001 between groups (delta values).

^#^ *P* < 0.05 versus placebo, ^##^ *P* < 0.001 versus placebo.

^¤^ *P* < 0.05 versus testosterone.

^§^ *P* < 0.05 versus training + placebo.

ST: strength training.

TT: testosterone treatment.

LBM: lean body mass.

SAT: subcutaneous adipose tissue.

Visceral adipose tissue was unchanged during all interventions, and these results are omitted from the table.
